# A Culturally Tailored Diabetes Self-Management Education Program With Mobile Health Integration for Chinese Americans With Type 2 Diabetes: Development and Pilot Evaluation Study

**DOI:** 10.2196/77372

**Published:** 2026-02-03

**Authors:** Bin Xie, Yawen Li, Wei-Chin Hwang, Zhongzheng Niu, Xiaomeng Lei, Ruizhi Yu, Yvonne Lai, Tiffany Fong, Yunsheng Ma

**Affiliations:** 1 Claremont Graduate University Claremont, CA United States; 2 California State University, San Bernardino San Bernardino United States; 3 Claremont McKenna College Claremont United States; 4 University at Buffalo, State University of New York Buffalo, NY United States; 5 University of Southern California Los Angeles, CA United States; 6 University of Nevada, Las Vegas Las Vegas, NV United States; 7 Center for Disease Control and Prevention Atlanta, GA United States; 8 University of Massachusetts Lowell Worcester, MA United States

**Keywords:** Chinese Americans, type 2 diabetes self-management, mobile technology, WeChat, cultural tailoring

## Abstract

**Background:**

Although progress has been made in improving the efficacy of Diabetes Self-Management Education (DSME) programs, there remains a dearth of research on culturally adapted, evidence-based DSMEs for Chinese Americans (CAs) with type 2 diabetes.

**Objective:**

Through collaborative partnerships with 2 large community recreation centers and the AHMC Hospital Network in San Gabriel Valley, California, we developed and pilot-tested a culturally tailored DSME program with integrated mobile health (mHealth) technology, entitled Culturally Appropriate Strategies for Chinese Americans with Diabetes (CASCADe).

**Methods:**

The CASCADe program utilized a combined, theoretically driven, and community-participatory approach and was developed based on information gleaned from focus groups, semistructured interviews, and a questionnaire survey conducted among CA patients with diabetes, physicians, and nurses, as well as from extensive literature reviews of evidence-based program curricula. A single-group pre- and posttest design with a 3-month study period was then employed to assess the program’s preliminary efficacy. The study protocols were registered on ClinicalTrials.gov.

**Results:**

The CASCADe program consisted of (1) a home visit in the first month for training in monitoring device use and WeChat app (a mobile instant-messaging platform widely used in the Chinese population) usage, as well as for acquiring family support; (2) 8 weekly sessions over the following 2 months, delivered in a combined format of group classes, games, group exercises, videos, and discussions; and (3) WeChat follow-up involving education tips, monitoring data summaries, and group discussions after each of the 8 weekly sessions. Topics covered in the weekly sessions included recognition of diabetes and its complications, risk factors, nutrition knowledge, dietary practices, exercise, behavioral self-monitoring, medication adherence, and stress management. The monitoring system used a smartphone to coordinate cloud-based data transmission from a set of wireless devices to capture daily monitoring data on physical activity, body weight, blood pressure, and blood glucose levels. Behavioral self-monitoring was further facilitated by the WeChat app, which provided daily messages related to the diabetes education curriculum; weekly summary reports of monitoring data; feedback; bidirectional 1-on-1 communication between intervention providers and participants; and group discussions among participants regarding readings and the implications of monitoring results. The pre- and postcomparison from the 3-month pilot trial showed a significant reduction in glycated hemoglobin (HbA1c; 7.48 vs 7.09, *P*=.03), with all but 1 participant demonstrating a reduction and 7 out of 12 (58%) achieving a >0.5 decrease in HbA1c. Significant improvements were also observed in self-efficacy in diabetes management (6.59 vs 8.01, *P*=.003), quality of life (3.21 vs 3.69, *P*=.005), and stress-coping skills (3.18 vs 3.74, *P*=.01) at 3 months after baseline among CA patients with type 2 diabetes.

**Conclusions:**

Our pilot study demonstrated the feasibility of implementing the CASCADe program among CAs to improve diabetes self-management skills and yielded promising results, warranting further evaluation in a larger randomized trial.

**Trial Registration:**

ClinicalTrials.gov NCT04737499; https://clinicaltrials.gov/ct2/show/NCT04737499

## Introduction

### Background

Asian Americans are proportionately the fastest-growing group in the United States, with Chinese Americans (CAs) representing the largest ethnic subgroup [1]. A report from the Diabetes Study of Northern California (DISTANCE) revealed that 104,000 (8.2%) out of 1.3 million CAs were diagnosed with type 2 diabetes mellitus (T2DM) [2]. CAs have less access to self-management education for diabetes compared with other ethnic groups [3,4], due to barriers such as language, difficulty with lifestyle transitions, financial constraints, and lack of health insurance [5-7]. A Pew Research Center [8] analysis of the American Community Survey reported that only 42% of foreign-born CAs could speak and write English well. Even when self-management programs are translated into Chinese, most are based on Western culture and lifestyle, including food choices [9]. CAs have found it difficult to manage their disease in the US context due to cultural and dietary differences [7,10]. Efforts to culturally adapt Diabetes Self-Management Education (DSME)—a key component in achieving effective glycemic control and an important part of clinical management [11]—have shown promising results in improving glycemic control and reducing cardiovascular morbidity and mortality among Whites, Latinos, and African Americans [12]. However, very limited efforts to culturally adapt evidence-based DSME programs have been made for CAs with T2DM [13-15], leading to scientifically untested assumptions that existing diabetes programs will be equally effective for CAs as for other ethnic groups.

One of the most important components of all DSME programs is patients’ self-monitoring of blood glucose levels, use of antidiabetic medications, and other lifestyle changes, such as diet and exercise. Traditional tools (eg, paper and pencil) used for diabetes self-monitoring and tracking lifestyle changes have low compliance, which affects the sustainability of intervention effects. To better support daily diabetes management, mobile health (mHealth) technologies (eg, SMS text messaging, phone calls, smartphone apps, and wearable devices) provide a convenient and effective platform for promoting healthy behaviors and improving blood glucose control [16-18]. Moreover, mHealth can facilitate active collaboration between patients and health care providers.

### Objectives

To address these gaps in the field, our research team undertook a 2-phase process to develop and pilot test a culturally tailored DSME program integrating mHealth technology, entitled Culturally Appropriate Strategies for Chinese Americans With Diabetes (CASCADe). Phase I used a mixed methods approach involving focus groups, semistructured interviews, and a questionnaire survey among CA patients with T2DM and health care providers (physicians and nurses), as well as extensive literature reviews of evidence-based DSME curricula. Information collected in phase I was used to inform the development of the CASCADe program curriculum. In phase II, a single-group pre- and posttest design with a 3-month study period was employed to pilot test the program’s preliminary efficacy. We hypothesized that participants receiving the CASCADe intervention would show a significant reduction in glycated hemoglobin (HbA_1c_) and significant improvements in self-efficacy, quality of life, and stress-coping skills for diabetes self-management at the end of 3 months, compared with their pretest levels.

## Methods

### Ethical Considerations

All study protocols for sample recruitment, data collection, intervention delivery, and evaluation were reviewed and approved by the Institutional Review Board of Claremont Graduate University (Institutional Review Board approval numbers 2522 and 2523). The protocol for the CASCADe intervention delivery and evaluation (ie, phase II study) was also registered on ClinicalTrials.gov (NCT04737499). We used active informed consent for both phase I and phase II studies. Participants were provided with an informed consent form that included information about the study purpose and background, procedures, risks or discomforts, extent of confidentiality (eg, each participant was provided with a unique study ID and study data were deidentified), benefits, incentives, and contact information for the primary investigator and the Claremont Graduate University Institutional Review Board. Each participant in phase I and phase II received US $10 as an incentive for participation. Phase II participants received an additional US $40 (US $5 for each of the 8 group-class sessions) for their time and travel. Compensation was provided as gift cards from 99 Ranch Market, the most popular Chinese grocery store in Los Angeles. In addition, various small gifts were offered as incentives during the education sessions. These gifts, such as a foot inspection mirror, measuring spoon, food scale, and resistance bands, were low in monetary value but served as strong motivators for participants to attend sessions and apply what they learned at home.

### Community Engagement

We established collaborative partnerships with 2 local senior recreation centers (ie, Langley Senior Citizen Center in Monterey Park City and Joslyn Adult Recreation Center in Alhambra City) and AHMC Healthcare Inc. Each recreation center serves over 800 seniors, the majority of whom are CAs. These centers regularly provide a variety of recreational, social, educational, and cultural services and programs (eg, daily lunch programs, fitness programs, free health screenings, and case management services) to seniors in the local communities. AHMC Healthcare Inc is a hospital corporation based in the Greater San Gabriel Valley, California, with a mission to provide health care services across primary and tertiary care. It is one of Southern California’s premier health care systems and operates a network of 6 hospitals, treating over 200,000 patients annually across more than 1100 beds. AHMC has a patient education center that includes classrooms, a large kitchen, and a laboratory. The Langley Center has classrooms, a computer laboratory, a large kitchen, and a gym with exercise facilities. The Joslyn Center has conference rooms and classrooms. Both centers and the AHMC Hospital Network helped recruit participants by distributing recruitment flyers, referring patients to the project, and providing space (eg, classrooms) and facilities for data collection and intervention delivery. AHMC Healthcare Inc also helped recruit physicians and nurses for interviews during the formative research phase and provided funds through its affiliated AHMC Health Foundation to support community diabetes screenings. Partial funding for our pilot program came from the AHMC Healthcare Foundation, which was deeply invested in the program.

### Participant Recruitment

The cities of Monterey Park and Alhambra, located in the San Gabriel Valley, each have a CA population of over 35% of their total residents on average. The AHMC Hospital Network provided us with T2DM statistics from its patient information database in 2017. Therefore, in both phase I and phase II studies, we recruited participants through clinic referrals within the AHMC Hospital Network and by distributing flyers at Chinese New Year events and senior recreation centers in Monterey Park and Alhambra.

For phase I formative research, 23 CA patients with T2DM were recruited through flyers distributed at AHMC Healthcare–affiliated clinics and at Chinese New Year events. All patients were (1) previously diagnosed with T2DM and managing their diabetes through diet, oral hypoglycemic agents, insulin, or a combination of these; (2) residents of the San Gabriel Valley; and (3) Mandarin speakers. None had other self-reported or clinically diagnosed physical or mental illnesses (eg, cardiac conditions, cancer, or major depression). In addition, 3 physicians and 3 nurses from AHMC Healthcare–affiliated clinics participated in the semistructured interviews.

For phase II (pilot test of the CASCADe program curriculum), we screened and recruited participants at AHMC-affiliated clinics, Chinese New Year events, and senior centers in Monterey Park and Alhambra, CA. Eligible criteria for participation included (1) diagnosis of T2DM; (2) managing diabetes with diet, oral hypoglycemic agents, insulin, or a combination of these; (3) confirmed HbA_1c_ level ≥6.5% and <10% (HbA_1c_≥10% indicating complicated diabetes requiring extensive medication); (4) 21-75 years old; (5) residents of the San Gabriel Valley; (6) Mandarin speakers; and (7) willing to use the smartphone-based monitoring devices provided by the study. Exclusion criteria included individuals who were unable or unwilling to give informed consent; planned to move out of the area within the study period; were pregnant, planning pregnancy, or breastfeeding within the next 6 months; had conditions that inhibited movement (eg, unable to walk unaided or unable to walk a quarter mile without stopping); or had significant physical disability or conditions that prevented in-person participation. Fourteen participants were enrolled in the study from 2017 to 2018.

### Measures in Phase I and Phase II of the Study

#### Focus Group Discussions and Semistructured Interviews in Phase I of the Study

The goals of the focus group discussions with CA patients with T2DM were to gather their experiences and opinions on diabetes care practices (eg, dietary and physical activity behaviors, glucose monitoring, and medication compliance), and to assess the feasibility of smartphone use, text and picture messaging patterns, and perceived benefits of such technologies in enhancing diabetes management. Focus group discussions were conducted in Mandarin and included semistructured, open-ended questions designed to promote reflection and discussion, beginning with general topics and gradually moving to more specific issues. Semistructured interviews were also conducted with 3 physicians and 3 nurses/health educators to understand usual patient care, and the challenges and barriers faced when working with CA patients with T2DM. Interviews were conducted in either Mandarin or English, depending on participant preference, and followed the same structure as the focus groups, using open-ended questions progressing from general to specific topics. Audio recording consent was obtained from all participants before the sessions.

#### Questionnaire Surveys in Both Phase I and Phase II of the Study

In both phase I and phase II, participants completed a questionnaire developed based on our literature review. Detailed information on psychosocial and behavioral measures is provided in Table 1. A comprehensive translation and back-translation process was used for existing questionnaires that had not been previously translated into Chinese. The questionnaire was administered using Qualtrics, LLC.

**Table 1:** Psychosocial and behavioral measures.

Instrument/standardized measure	Scale information	Sample item
Diabetes Quality of Life [19]	15 items (1-5 Likert Scale^a^); α=.85	How satisfied are you with your current diabetes treatment?
Diabetes Distress Assessment [20-22]	15 items (1-6 Likert Scale^b^); α=.902	I feel that diabetes is taking up too much of my mental and physical energy every day.
Diabetes Management Self-Efficacy Scale [23]	8 items (1-10 Likert Scale^c^); α=.93	I am able to choose different foods and maintain a healthy eating plan when I am eating out or at a party.
Diabetes Knowledge Test [24,25]	14 items (multiple choices); α=.62	Which of the following is highest in carbohydrate? a. Baked chicken b. Swiss cheese c. Baked potato d. Peanut butter.

^a^For the Diabetes Quality of Life Scale, the original response options ranged from 1 to 5, representing very satisfied, moderately satisfied, neither satisfied nor dissatisfied, moderately dissatisfied, and very dissatisfied. The scale was reverse-coded to range from 5 to 0, with higher scores indicating a higher quality of life.

^b^For the Diabetes Distress Assessment Scale, the original response options ranged from 1 to 6, corresponding to not a problem (1-2), moderate problem (3-4), and serious problem (5-6). The scale was reverse-coded to range from 5 to 0, with higher scores indicating greater stress-coping skills.

^c^For the Diabetes Management Self-Efficacy Scale, responses ranged from 1 to 10, representing not at all confident to totally confident, with higher scores indicating greater self-efficacy.

#### Measures to Assess the Preliminary Efficacy of the CASCADe Program in Phase II of the Study

The primary outcome used to evaluate the preliminary efficacy of the CASCADe program curriculum in phase II was HbA_1c_ level, assessed using the A1CNow+ point-of-care assay with a finger-prick blood sample collected by trained research assistants. This assessment method has demonstrated excellent accuracy (99% on average) in validation studies comparing A1CNow+ results with National Glycohemoglobin Standardization Program–certified laboratory reference results. HbA_1c_ assessment using finger-prick sampling has also been employed in other clinical trials evaluating diabetes treatment outcomes [14,26,27]. As suggested by the American Diabetes Association, HbA_1c_ reflects mean blood glucose concentrations over approximately the previous 8-12 weeks and provides a better indication of long-term glycemic control than fasting blood glucose [28,29]. Secondary outcomes included psychosocial and behavioral measures (listed in Table 1), as well as blood pressure, body weight, height, and waist circumference, which were measured by research assistants following standard protocols. Both primary and secondary outcomes were assessed at baseline (pretest) and at the end of the 3-month intervention (posttest).

In addition, participants’ medical history, including years since diagnosis of T2DM, medication use, and diabetes complications (ie, eye problems; loss or decrease of sensation in the feet or legs; kidney problems; heart failure, angina, or heart attack; foot ulcer or infection; stroke; amputation; sexual problems; and symptoms of hypoglycemia), was self-reported. Research assistants also reviewed the use of any oral or injectable antidiabetic medications for each participant. Finally, to assess the acceptability of the CASCADe program curriculum, study dropout and intervention retention (eg, group class attendance, self-monitoring completion rate) were evaluated.

### Data Analysis

Recordings of focus groups and semistructured interviews were transcribed and analyzed. An inductive thematic analysis was employed, allowing themes to emerge from the data rather than being fitted into a preexisting coding frame. This approach provided rich, detailed insights into patients’ and health professionals’ experiences and perspectives. Transcripts were repeatedly reviewed, coded iteratively, and grouped into major and minor themes by 2 independent student research assistants (ZN and XL), and were further reviewed and finalized by supervised researchers (ie, YWL and WCH), both of whom have extensive expertise and experience in qualitative data analysis. Discrepancies were resolved through discussion and consensus. Participants were not involved in reviewing the transcriptions or final qualitative results. SAS (version 9.4; SAS Institute) was used to manage and analyze all quantitative data. Descriptive analyses were conducted for means, SDs, frequencies, and proportions. Paired *t* tests (1-tailed) and the nonparametric Wilcoxon signed rank test were used to evaluate the preliminary efficacy of the CASCADe program for both primary and secondary outcomes.

## Results

### Phase I Formative Research

We conducted 5 focus group discussions with 23 patients (14 females and 9 males, aged 55-83 years) with T2DM living in the San Gabriel Valley. The discussions highlighted several challenges CAs encountered in diabetes management, including inadequate communication with physicians or other health practitioners (eg, clinical dietitians), insufficient culturally appropriate health education and materials, and limited access to training to optimize diabetes self-management. The most significant challenges reported were dietary control and blood glucose monitoring. Most patients described usual primary care as consisting mainly of medications and occasional educational brochures or pamphlets provided by clinics or community centers. Some had attended health fairs or occasional health seminars hosted by the city government, community centers, or hospitals. Although some patients sought diabetes self-management advice from health care providers, many reported learning diabetes care tips primarily from social media, friends, and family members, who may be misinformed and provide ineffective recommendations (eg, “eating less food and drinking more water”).

Table 2 presents general sample characteristics and diabetes-related psychosocial measures. On average, participants had a diabetes history of 18 years, and all were first-generation immigrants—17 (74%) originally from mainland China, with the remainder from Taiwan, Hong Kong, and Vietnam. Participants reported an average sum score of 9.96 (SD 2.18) out of 14 on diabetes knowledge questions, an average score of 2.93 (SD 1.17) on a 1-6 Likert Scale for diabetes distress, 2.61 (SD 0.70) on a 1-5 Likert Scale for diabetes quality of life, and 6.73 (SD 1.82) on a 1-10 Likert Scale for diabetes self-efficacy. These results indicated a need to improve diabetes knowledge and to address the psychological impact of diabetes on CAs.

In addition, we interviewed 3 physicians and 3 nurses/health educators regarding the usual care patients received and the challenges and barriers encountered when working with CA patients with T2DM. Clinician interviews reaffirmed the needs identified by patients and further highlighted frustrations faced by health professionals—particularly the lack of culturally appropriate diabetes educational materials available for CA patients.

Our formative research highlighted the needs and directions for developing a culturally tailored DSME program for CAs. Such programs have the potential to improve diabetes self-management by enhancing communication, cultural awareness, and competencies, as well as by changing dietary and behavioral practices. Feedback from the focus group discussions and the questionnaire survey provided valuable insights into target areas for our culturally responsive curriculum and the benefits of integrating smartphone technology to support self-management.

**Table 2:** General sample characteristics (N=23).

Characteristics			Values
Age (years), mean (SD)			69.43 (8.35)
Gender, male, n (%)			9 (39)
**Place of birth, n (%)**			
	Mainland China		17 (74)
	Taiwan		3 (13)
	Hong Kong		1 (4)
	Other places outside the United States		2 (9)
Length of stay in the United States (years), mean (SD)			21.79 (15.10)
**Educational attendance, n (%)**			
	Elementary/middle school		4 (17)
	High/alternative high school		8 (35)
	Some college		3 (13)
	College		5 (22)
	Graduate		3 (13)
**BMI (kg/m^2^), mean (SD)**			
	Female		23.71 (2.80)
	Male		26.56 (2.60)
Age (years) at diagnosis, mean (SD)			51.64 (13.12)
Years with diabetes, mean (SD)			17.18 (15.53)
Using insulin (yes), n (%)			4 (17)
Using medications (yes), n (%)			21 (91)
Family history (yes), n (%)			16 (70)
Hypertension (yes), n (%)			17 (74)
Smartphone (yes), n (%)			14 (61)
WeChat account (yes), n (%)			7 (30)
Diabetes knowledge, mean (SD)			9.96 (2.18)
**Diabetes stress, mean (SD)**			
	Overall		2.93 (1.17)
	Emotional burden		2.90 (1.27)
	Physical distress		3.08 (1.46)
	Regimen distress		3.19 (1.21)
	Interpersonal distress		2.38 (1.37)
Diabetes—quality of life, mean (SD)			39.22 (10.43)
Diabetes—self-efficacy, mean (SD)			6.73 (1.82)
**Diabetes support, mean (SD)**			
	Emotional support		17.15 (5.64)
	Advice support		17.40 (7.16)
	Information support		17.56 (6.51)
**Acculturation, mean (SD)**			
	Overall		1.85 (0.38)
	Language		1.35 (0.57)
	Identity		2.09 (0.58)
	Friendships		1.39 (0.53)
	Behavior		1.91 (0.58)
	Generation		1.10 (0.35)
	Attitudes		2.17 (1.11)
**Brief symptoms inventory, mean (SD)**			
	Somatic		1.80 (0.63)
	Obsessive compulsive		2.09 (0.52)
	Interpersonal sensitivity		1.70 (0.58)
	Depression		1.58 (0.48)
	Anxiety		1.54 (0.54)
	Hostility		1.70 (0.60)
	Phobic anxiety		1.47 (0.48)
	Paranoid ideation		1.91 (0.77)
	Psychoticism		1.37 (0.36)

### Phase I CASCADe Program Curriculum

Besides the mixed methods of focus groups, semistructured interviews, and a questionnaire survey, we conducted an extensive and comprehensive review of the literature on both nonculturally tailored and culturally tailored diabetes self-management programs to evaluate the strengths and weaknesses of extant evidence-based interventions, as well as to inform the creation of a bank of psychosocial and behavioral measurements. After this extensive evaluation, we decided to adapt the CASCADe program prototypes from the evidence-based curricula recommended by the American Diabetes Association [30] and the International Diabetes Federation [31]. Rather than focusing on surface-structure adaptations (eg, translation of materials and delivery in Mandarin Chinese), our program used a community-participatory framework (eg, focus groups with patients and providers) to identify areas for deep structural adaptations (eg, incorporation of cultural beliefs, barriers, values, communication styles, relationship building and support, understanding of Traditional Chinese Medicine and its impact on adherence, and culturally syntonic health and dietary behaviors and practices) [32,33]. The curriculum design was closely aligned with a theoretical framework comprising behavioral strategies and skills (eg, goal setting, self-efficacy, positive reinforcement, and peer support) drawn from the Planned Behavior Theory [34], the Social Cognitive Theory, the Social Learning Theory [35,36], and the Self-Regulation Theory [37]. The CASCADe curriculum started with a home visit in the first month to provide training on the use of monitoring devices and the WeChat (Tencent Holdings Limited) app, as well as to acquire family support. We acknowledge that participants’ educational background and technological experience would help understand their overall responses to the questions and evaluations, as individuals’ technical expertise can affect the adoption and integration of this program. In the following 2 months, 8 sessions (90 minutes each) using a combined format of group classes, educational games, group exercises, educational video viewing, and discussions were implemented. Throughout the entire study period, participants were instructed to use the monitoring devices for behavioral self-monitoring and to receive WeChat follow-ups, including educational tips, summaries and feedback on monitoring data, bidirectional 1-on-1 discussions between intervention providers and participants, as well as group discussions among participants.

Topics of the culturally adapted education sessions included recognition of diabetes and its complications, risk factors, dietary practices, exercise, behavioral self-monitoring, medication adherence, and stress management. Highlights of the session content are outlined in Table 3. The group-class format, as a major intervention delivery approach, has been widely used in other DSME programs for American adults [15] and is considered culturally syntonic for CAs from a collectivistic cultural background who value education. During the education sessions, we adopted this approach to enhance participants’ learning by better attaching shared meaning to their experiences [38] and practice [39]. Session activities included class lectures with presentations, case studies, watching short video episodes and clips, storytelling of personal experiences (eg, stress coping), playing games (eg, ordering a meal plan through a mock dining-out experience at a Chinese restaurant), sharing tips on healthy cooking and smart grocery shopping, and reading food labels. At the beginning of each group session, participants were invited to follow an instructional video we created for a 15-minute workout using stretching bands, followed by a short video case study introducing the topic of that session. For example, the video episode for group session 1 included 2 cases addressing symptoms and diagnosis of T2DM and methods for checking complications. At the end of each group session, a factsheet summarizing key concepts in bullet points was reviewed, and participants were quizzed on the material covered in that session. We developed 2 bilingual handbooks, 1 for health educators and 1 for participants.

**Table 3:** Outline of the CASCADe^a^ program by session.

Session topics	Highlights of the session
Group session 1: Recognition of Diabetes and Complications	Icebreaker game Understand the basics of diabetes in both Traditional Chinese Medicine and Western Medicine (eg, diagnosis, symptoms, complications) context Identify organs affected by diabetes complications using anatomy graphs Sharing personal experience and goal setting Discuss the pros and cons of Chinese norms, beliefs, and attitudes toward diabetes and treatment
Group session 2: Diabetes Risk Factors	Understand diabetes-related risk factors (eg, poor diet, physical inactivity, family history, obesity, hyperlipidemia, hypertension, smoking) and guidelines and assessments Measurement demonstration (eg, BMI, blood pressure, blood glucose) and interpretation Information on sources for health services
Group sessions 3 and 4: Nutrition and Dietary Practice	Nutrition misconceptions and food myths in the Chinese American community and social media Create daily carbohydrate gram goals Glycemic index, glycemic load, and identify high and low glycemic foods Nutrition labels Mock dining at restaurants Tips on healthy cooking and smart grocery shopping Portion size guides and healthy eating guidelines Set diet-related goals, self-monitoring, and self-reinforcement
Group session 5: Exercise	Physical activity guidelines Misconceptions and myths of physical activity Chinese norms and beliefs of exercise and diabetes Introduce popular Chinese exercises (eg, Tai-Chi, square dancing) Incorporate exercise into daily routine life Discuss the benefits, safety, and barriers of exercising Set physical activity goals and self-monitoring reinforcement
Group session 6: Self-Monitoring	Importance of self-monitoring blood glucose, body weight, blood pressure, culturally syntonic physical activity, and eating practices How to read and interpret results, how factors may affect results, and how to keep adequate and appropriate monitoring records Importance of reinforcement, peer and family support, and use of technology on self-monitoring adherence
Group session 7: Medication Adherence	Discuss types of commonly used antidiabetic medicine such as sulfonylureas, metformin, rosiglitazone, pioglitazone, or insulin, or any combination; safety on dosage change; side effects of common antidiabetic medications, including hypoglycemia episodes; recognizing typical symptoms of hypoglycemia and self-management; and Chinese herbal medicine and myths of alternative medicine (eg, Qigong and acupuncture) Importance of antidiabetic medication adherence Read the prescription label Review medication adherence-related goals, self-monitoring, and self-reinforcement
Group session 8: Stress Management and Social Support	How to assess stress, anxiety, and depression Tips on culturally effective stress-coping strategies Understand the importance of social support, assess the availability of support and service resources, build a support network, balance support and obligation, and navigate a cultural minefield in seeking and receiving support

^a^CASCADe: Culturally Appropriate Strategies for Chinese Americans with Diabetes.

**Figure 1:**
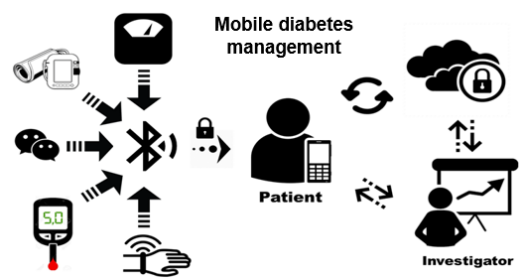
Mobile diabetes monitoring and management system.

During the 3-month study intervention period, each participant was provided with a mobile technology–assisted self-monitoring system, which was a smartphone-based, integrated health monitoring system with clinically validated devices manufactured by iHealth Labs Inc [40,41]. The system consisted of a smartphone and a set of Bluetooth-enabled wireless devices that provided continuous cloud-based data synchronization, including (1) a glucose meter for monitoring glucose levels; (2) a blood pressure cuff for assessing blood pressure and heart rate; (3) a smart weight scale for measuring body weight and BMI; and (4) a wrist band for tracking daily activity and sleep patterns (Figure 1). The iHealth MyVitals app, a central mobile phone–based platform, connected all devices via Bluetooth and automatically uploaded users’ data to a secure cloud environment, allowing access by authorized personnel (eg, lifestyle coach, researcher). In addition, the WeChat app, a mobile instant messaging platform widely used in the Chinese population, was used to facilitate behavioral self-monitoring by delivering daily messages, educational tips, and supplementary readings related to the diabetes education sessions, as well as weekly summary reports of monitoring data. The instant messaging function also supported bidirectional 1-on-1 discussions between each participant and the health educator regarding the implications of monitoring results and reinforcement strategies, as well as online peer-based group discussions and social networking among participants to reinforce lifestyle changes and provide peer support. We also created a bank of WeChat articles corresponding to each classroom instruction session, along with an SMS text message bank (including graphs and short video clips) containing motivational messages on various diabetes management topics. Comprehensive training on the use of smartphones, the WeChat app, and Bluetooth-enabled behavioral self-monitoring devices, as well as privacy protection, was provided during the first home visit, with ongoing support (eg, Q&A and troubleshooting) offered by trained personnel and a hotline throughout the study period. In this pilot study, self-monitoring devices and smartphones were provided to participants. The smartphones were preloaded with all required apps, including WeChat and iHealth MyVitals, to support daily communication and self-monitoring data management. To further facilitate participants’ use of mHealth technology, the phones were also preloaded with short instructional videos demonstrating the procedures for using each self-monitoring device, as well as a 15-minute stretching band exercise video used in the group class sessions. In addition to technology instruction guides, a color-illustrated, step-by-step, laminated guide was provided to assist participants in learning and using the mobile technologies. This was supplemented by a short instructional video clip accessible through participants’ WeChat accounts. For data acquired through the mobile self-monitoring system (eg, body weight, blood pressure, average pulse rate, blood glucose, and physical activity measured as walking steps and calculated minutes of moderate-vigorous and vigorous activity), descriptive analysis algorithms (eg, means, SDs, frequencies, and proportions presented in tables and charts) were developed to generate weekly summary reports for both participants and investigators. All program content, technical interventions, and clinical discussions were conducted in Mandarin.

To compensate intervention participants for their time and travel, we provided incentives in the form of a US $10 gift card for each class session and an additional US $5 for WeChat and group chat participation. In addition, training tools (eg, a foot inspection mirror, measuring spoon sets, a pocket-size food scale, a stretching band, and educational posters) used during the class sessions were provided as small gifts to incentivize attendance and participation.

### Preliminary Efficacy of the CASCADe Program

We recruited 14 participants to join the program; however, 2 dropped out (1 returned to mainland China and the other was unavailable during the scheduled class sessions). Of the remaining 12 participants, 7 (58%) were female, and 5 (42%) were male. The mean age was 65 years (range 54-72 years). Half of the participants were born in Mainland China, 4 were from Taiwan, and 2 were from other locations outside the United States. A total of 5 (42%) participants had completed elementary or middle school, 3 (25%) had completed high school, 1 (8%) had graduated from a 4-year college or university, and 3 (25%) had attended some graduate or professional school. The average duration since diabetes diagnosis was 15 years. One participant had been diagnosed for less than 10 years, 9 had been diagnosed for 10-20 years, and 2 had been diagnosed for 20 years or more. Most participants had hypertension (n=9, 75%); 2 (17%) had cancer, and 1 had no physical disease. None of the participants had a mental disease.

A summary of pre- and posttest results for biomarkers and psychological indicators is presented in Table 4. Preliminary pre-post comparisons among participants showed significant improvements in HbA_1c_ levels (7.48 vs 7.09, *P*=.03; all but 3 participants reduced HbA_1c_ levels without changes in antidiabetic medication, and 7/12, 58%, participants achieved a >0.5 reduction), diabetes knowledge (7.67 vs 9.0, *P*=.04), self-efficacy (6.59 vs 8.01, *P*=.003), and quality of life (3.21 vs 3.69, *P*<.001) at 3 months after baseline (Table 4). A significant improvement was also observed in the average score for stress-coping skills (3.18 vs 3.74, *P*=.01), which was measured using a reverse-coded Diabetes Distress Scale (ie, the original Likert Scale of 1-6 was reverse coded to 5-0, with higher scores indicating better stress-coping skills). No significant changes were observed in body weight (*P*=.45) or BMI (*P*=.50).

**Table 4:** Summary of pre-post test results on biomarker and psychological indicators.

Outcome	Pretest, mean (SD)	Posttest, mean (SD)	Pre-post difference, mean (SE)	*P* value (1-tailed)
HbA_1c_^a^	7.48 (0.69)	7.09 (1.25)	–0.38 (0.19)	.03
BMI	26.51 (1.82)	26.51 (1.84)	–0.0006 (0.25)	.50
Waist circumference	96.02 (7.40)	93.87 (5.73)	–2.16 (1.20)	.05
Stress coping^b^	3.18 (1.38)	3.74 (0.91)	0.56 (0.22)	.01
Self-efficacy	6.59 (1.76)	8.01 (1.37)	1.37 (0.46)	.007
Quality of life	3.21 (0.77)	3.69 (0.55)	0.48 (0.14)	.002
Diabetes knowledge	7.67 (2.27)	9.0 (1.81)	1.33 (0.71)	.04

^a^HbA_1c_: glycated hemoglobin.

^b^The Stress-Coping Scale was the reverse-coded Diabetes Distress Scale (ie, the original Likert Scale of 1-6 was reverse-coded to 5 to 0, with a higher score indicating a higher level of stress coping skills).

**Figure 2:**
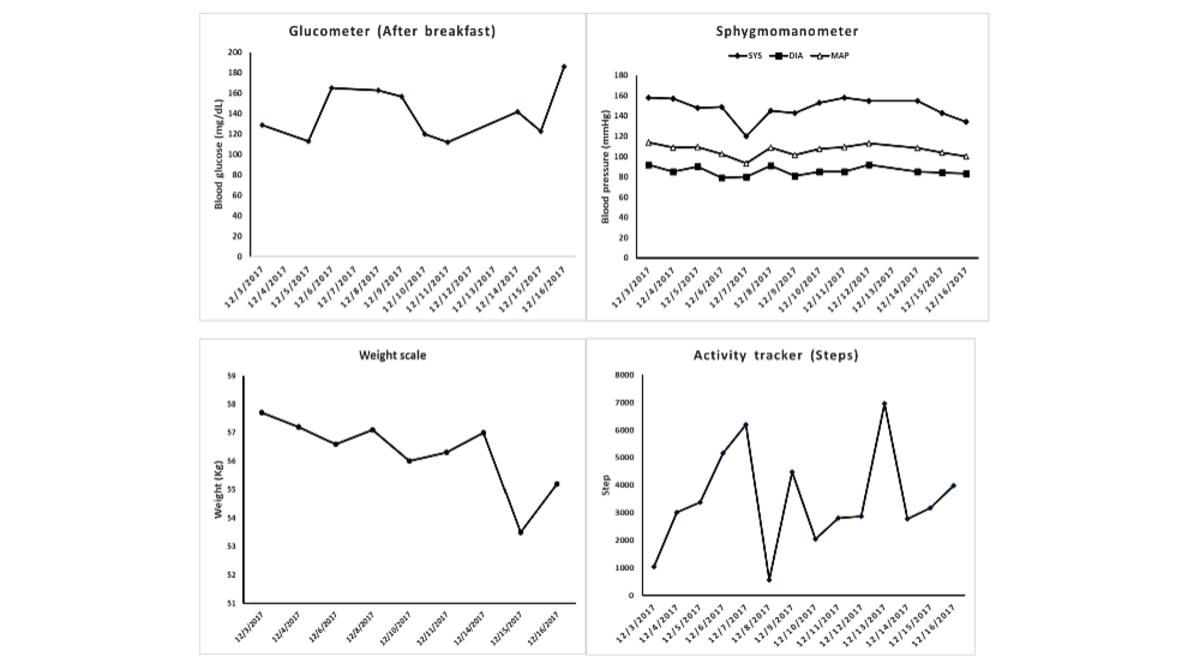
Fig 2. Bi-Weekly Self-Monitoring Chart for One Participant.

Tables 5 and 6 present a summary of the evaluation of the intervention program components and adherence. All but 3 participants attended all 8 group class sessions, and those 3 missed only 1 session during the study period. All participants used smartphones, WeChat, and mHealth devices for self-monitoring. All participants reported being “very satisfied” with the CASCADe program, regarded the program as “very culturally responsive to (their) needs,” were adherent to self-monitoring using the smartphone-based mobile monitoring system, and indicated that WeChat was “very helpful” or “helpful” for engaging in group discussions; posting and receiving health tips, reminders, and reinforcements; and reading daily articles and personal weekly summary reports of behavior monitoring; 9 out of 11 (82%) participants endorsed the smartphone-based self-monitoring system as “acceptable,” “easy to use,” or “very easy to use.” The only complaint related to familiarity with participants’ own glucose meters, as some were less enthusiastic about using the new glucose meter provided, which was integrated into the smartphone-based mobile monitoring system via Bluetooth. An example of a biweekly self-monitoring chart from one participant is illustrated in Figure 2.

**Table 5:** Summary of the evaluation of the intervention program.

Evaluation components		Positive feedback^a^
Overall intervention, n (%)		11 (100)
**Class sessions, n (%)**		
	Health educational video	11 (100)
	Resistance band exercise	11 (100)
	Instruction delivery	11 (100)
	Quiz	10 (91)
**Self-monitoring instruments, n (%)**		6 (55)
	Weight scale	
	Glucometer	6 (55)
	Sphygmomanometer	7 (58)
	Activity tracker	7 (58)
**WeChat, n (%)**		
	Daily tweets	10 (91)
	Personal weekly report	11 (100)
	WeChat group discussion	9 (82)

^a^n=11, 1 out of 12 participants did not attend the last course to complete the evaluation survey. Positive feedback: combination of “good” and “very good,” “helpful” and “very helpful,” “satisfied” or “like.”

**Table 6:** Summary of intervention adherence.

Intervention adherence		Participation rate^a^
**Class sessions, n/N (average percentage)**		
	Class attendance	91/96 (95)
	In-class quiz	93/96 (97)
Home visits, n (%)		12 (100)
WeChat article-related quiz, average percentage (SD)		Round 1: 0.87 (0.20); round 2: 0.65 (0.45); and average percentage: 0.79 (0.31)
**Self-monitoring instrument, average percentage (SD)**		
	Weight scale	0.46 (0.33)
	Glucometer	0.67 (0.46)
	Sphygmomanometer	0.70 (0.32)
	Activity tracker	0.65 (0.31)

^a^The participation rates between class attendance and in-class quizzes differed among 12 participants. The reason is that some participants who missed the course learned the material on their own at home and made up the previous quiz when they attended the next class session. Both participation rates were calculated as the number of class attendance or in-class quizzes divided by the product of 12 participants by the total number of class sessions (8 sessions) or in-class quizzes. The means and SDs were provided for adherence to the WeChat article-related quizzes and the self-monitoring instrument.

## Discussion

### Principal Findings

Through collaborative partnerships with 2 large community recreation centers and the AHMC Hospital Network in San Gabriel Valley, California, we developed and pilot-tested a culturally tailored DSME program with integrated mHealth technology, entitled CASCADe. Preliminary results from the pilot trial demonstrated a significant reduction in HbA_1c_, along with improvements in diabetes management knowledge, self-efficacy, quality of life, and stress-coping skills among CA patients with type 2 diabetes.

Although progress has been made in improving the efficacy of DSME programs, there remains a dearth of research on culturally adapted, evidence-based DSMEs for CAs with T2DM. Our research efforts are expected to help address the growing needs of CAs with T2DM by advancing knowledge of best practices in T2DM self-management. This is particularly important given the rapidly increasing prevalence of T2DM among CAs [2-4], making investment in such efforts more urgent than ever.

The CASCADe program used a combined, theoretically driven, and community-participatory approach to develop an evidence-based curriculum comprising 8 weekly sessions of health education and diabetes management. The monitoring system employed a smartphone to coordinate cloud-based data transmission from a set of wireless devices to capture daily monitoring data on physical activity, body weight, blood pressure, and blood glucose levels. The WeChat app was used to facilitate behavioral self-monitoring by delivering daily messages aligned with the diabetes education curriculum, weekly summary reports of monitoring data, reinforcement messages, and group discussions on readings and the implications of monitoring results. The CASCADe program was successfully piloted, and preliminary results indicated a significant reduction in HbA_1c_, along with improvements in diabetes management knowledge, self-efficacy, quality of life, and stress-coping skills among CA patients with type 2 diabetes.

Our study found results comparable to prior research on diabetes education programs for CAs. Culturally adapted programs for CAs reported in a recent systematic review included 2 conducted in San Francisco [13,14], 1 in Hawaii [15], and another that tested a linguistically and culturally tailored Diabetes Prevention Program among CAs with prediabetes in New York City [42]. All of these programs demonstrated improved treatment outcomes, including self-efficacy, diabetes knowledge, family support, diabetes distress, and quality of life. A significant reduction in HbA_1c_ was reported in all studies except 1 [13]. The preliminary efficacy observed in our study was similar to that of these diabetes educational interventions for CAs [13-15] and for other minority populations in the United States [43-45]. A key innovation of our intervention was the use of a culturally practical and integrative mHealth technology to enhance self-monitoring. The Bluetooth-enabled devices increased participants’ willingness to engage in diabetes self-management monitoring by automatically uploading tracking data to a secure cloud, rather than requiring maintenance of monitoring logs and manual data entry. This approach enabled investigators to provide immediate, personalized feedback to participants upon receipt of the data [46,47]. In addition, the use of a widely adopted CA social media platform, WeChat, expedited bidirectional communication between participants and researchers and fostered a peer-supported community that connected participants and facilitated mutual support [46,47]. Although the pilot nature of the study limited the sample size and relied on a less rigorous single-group pre-post quasi-experimental design, the feasibility and preliminary efficacy findings are promising.

### Limitations

Given the pilot nature of our study, several limitations should be considered when interpreting the findings. These include limited statistical power due to the small sample size; potential selection bias (eg, volunteer bias) arising from recruitment strategies that relied primarily on referrals through the AHMC Healthcare Network and participation via flyers distributed at senior centers and Lunar New Year events; and the use of a single-group pre- and posttest design for the preliminary evaluation of program efficacy. Future work would benefit from incorporating in-depth interviews with phase II participants to better understand the mechanisms through which the intervention produced its effects. In particular, such qualitative data could elucidate how psychological factors (eg, self-efficacy and stress-coping skills) shaped behavioral changes, as well as how cultural values and adaptations were perceived and integrated by participants during the intervention.

### Implications for Diabetes Education

Lastly, our pilot study demonstrated the feasibility of implementing the CASCADe program among CAs to improve diabetes self-management skills. The study yielded promising results and warrants testing in a larger randomized trial. We expect to maintain our research collaborations, continue providing health screenings and the CASCADe program to the community, and expand this translational research model—which integrates academic research with community health services—to other ethnic minority populations.

### Conclusions

Our pilot study demonstrated the feasibility of implementing a culturally tailored intervention program with mHealth integration among CAs to improve diabetes self-management skills.

## Abbreviations

CA: Chinese American
CASCADe: Culturally Appropriate Strategies for Chinese Americans with Diabetes
DISTANCE: Diabetes Study of Northern California
DSME: Diabetes Self-Management Education
HbA1c: glycated hemoglobin
mHealth: mobile health
T2DM: type 2 diabetes mellitus

## Data Availability

The datasets generated and analyzed during this study are available from the corresponding author (BX) upon reasonable request.
